# Gastroenterological disease detection using transformer-based medical imaging for sustainable healthcare

**DOI:** 10.1038/s41598-026-45222-9

**Published:** 2026-03-30

**Authors:** Tanzila Kehkashan, Maha Abdelhaq, Ahmad Sami Al-Shamayleh, Muhammad Abdullah, Raja Adil Riaz, Sharifah Sakinah Syed Ahmad, Abdelmuttlib Ibrahim Abdalla Ahmed, Adnan Akhunzada

**Affiliations:** 1 Faculty of Computing, Universiti Teknologi Malaysia, 81310 Johor Bahru, Malaysia; 2 Faculty of Information Technology, University of Lahore, Sargodha, 40100 Pakistan; 3 Department of Information Technology, College of Computer and Information Sciences, Princess Nourah bint Abdulrahman University, 11671 Riyadh, Saudi Arabia; 4 Department of Data Science and Artificial Intelligence, Faculty of Information Technology, Al-Ahliyya Amman University, Amman, 19328 Jordan; 5 Faculty of Artificial Intelligence and Cyber Security, Universiti Teknikal Malaysia Melaka, Durian Tunggal, Malaysia; 6 Computer Science Department, Faculty of Computer Science and Information Technology, Omdurman Islamic University, Omdurman, Sudan; 7 Department of Data and Cybersecurity, University of Doha for Science and Technology, Doha, 24449 Qatar

**Keywords:** Curated colon dataset (CCD), Detection subtle anomalies, Gastrointestinal diseases, Prediction patient outcome, Vision transformer (ViT-B16), Computational biology and bioinformatics, Diseases, Engineering, Gastroenterology, Health care, Mathematics and computing

## Abstract

Early detection of gastroenterological diseases significantly improves patient outcomes and reduces late-stage diagnostic burden, yet traditional CNN models show limitations in capturing complex patterns within medical imaging datasets, prompting investigation into transformer architectures like Vision Transformer (ViT). Application of the ViT technology in detecting gastroenterological diseases with the help of medical imaging has not been fully explored, despite the promising capabilities. In this paper, the effectiveness of the ViT-B16 structure for the identification of gastrointestinal abnormalities is considered using a combined dataset of Curated Colon Dataset and HyperKvasir Dataset (10,000 images across four classes), and compared with established methodologies. Our experimental results showed that ViT-B16 performed better when compared to alternative approaches; it achieved 99.5% classification accuracy compared to 99.1% by EfficientNetB5 and 97.1% by EfficientNetB2, with other supportive performance metrics including precision (99.4%), recall (99.4%), and F1-score (99.4%), AUC values ranged from 0.99 to 1.00 across all classes, reflecting very strong discriminatory power regarding disease classification tasks. These suggest that ViT-B16 has great potential for medical diagnosis applications, especially classification tasks in healthcare, where evidence-based decision-making and model interpretability are key considerations. The model also supports sustainable healthcare through computational efficiency and reduced diagnostic burden. However, there are several challenges that have not been addressed, including addressing ethical concerns about diagnostics, improving diagnostic accuracy for underrepresented disease classes, and validating the model across diverse clinical settings, which are essential directions for future research to continue developing gastroenterological disease-detecting techniques.

## Introduction

Gastrointestinal disorders represent a wide range of pathological conditions, from the esophagus to the rectum, posing a significant challenge to public health globally. Such medical conditions account for a substantial proportion of global disease burden and death rates, affecting countless individuals throughout diverse demographic groups and geographical areas^1^. The rising incidence of GI disorders, in addition to their complex pathophysiology, has made them a point of much concern in medical research and clinical practice^2^. It is also well known that the cost burden to healthcare systems from GI disease is in the billions of dollars annually .

In the entire spectrum of GI diseases, inflammatory bowel diseases, or IBDs such as Crohn’s disease and ulcerative colitis have become prominent because of their chronic nature and increasing prevalence all over the world as conditions of specific concern. The disorders are characterized by chronic inflammation of the gastrointestinal tract and arise in the form of abdominal pain, diarrhea, and loss of weight^3^ . Etiology of IBD is multifactorial based on genetic predisposition, environmental factors, and immune system dysregulation making classification challenging^4^. Recent advances in medical image analysis have demonstrated the potential of deep learning architectures across various diagnostic domains, including breast cancer classification, diabetic retinopathy detection, and brain tumor segmentation, establishing foundational principles applicable to gastroenterological imaging^5–7^. Another related factor to be concerned about is the rising incidence of gastrointestinal cancers, particularly colorectal cancer; hence, early detection and prevention strategies are a challenge in medical diagnostics^8^.

Deep learning techniques have increasingly been applied over the last couple of years to GI disease diagnosis and management^9^. Application of Convolutional Neural Networks has been promising in automating polyp detection in colonoscopy images and videos^10^. Recurrent neural networks have been used in the study of the timeseries nature of electronic health records in an attempt to predict the course of disease in IBD patients^11^. Even though promising studies have been seen relating to deep learning in GI medicine, further generalization of such studies is challenging due to small datasets, poor patient diversity, and interpretability challenges in such complex models . Most of the studies concentrate on single modalities, specifically either only on imaging data or only clinical data, which may miss important cross-modal interactions^12^. The application of attention-based mechanisms and pyramidal feature extraction has shown promising results in various medical imaging tasks, including liver tumor detection and prostate cancer segmentation, suggesting their potential utility in gastrointestinal disease classification^13,14^.

Despite the advances made in deep learning applications for GI diseases, there is still a deep sense that such an approach needs to have comprehensive and multi-modal integration of different types of data to better relate to diagnostic accuracy and appropriate treatment planning. The baseline paper positioned the possibility of combining imaging, genomic, and clinical data in GI disease management but strongly emphasized challenges in effective integration of such diverse data types^15^. The proposed Vision Transformer-based deep learning framework will bridge this knowledge gap by offering enhanced diagnostic accuracy for a range of GI diseases through improved medical image classification.

The objectives of this research are:

i. To compare the diagnostic performance of the ViT-B16 with that of traditional CNN approaches for gastrointestinal disease classification on the Curated Colon Dataset.
ii. To demonstrate the model’s potential for sustainable healthcare through efficient computation and reduced diagnostic workload.
iii. To determine if the ViT-B16 model might be utilized for the early detection of gastrointestinal disorders in a standard clinical diagnostic environment.

ViT models’ ability to recognize GI disorders is one great improvement in the field of medical diagnostics imaging. Previous models in the field of GI disorders used the most basic approaches to imaging pre-processing while utilizing transformers to learn features. In this work though, the focus was in changing the pre-processing to something better, which might enable a more significant shift in the trust worthiness of the diagnosis tasks when it comes to early disease detection. The work also reviewed the performances of various state of the art CNN architectures, particularly those that incorporated explainability which was a great contribution towards the clinical deployment of CNN architectures. This work is going to change the way images in gastroenterology diagnostics are reviewed which will be beneficial in early disease detection and for the overall outcome for the patients.

In Section Literature review, we summarize previous works which apply deep learning in the gastrointestinal tract; multi-modal learning; and techniques in diagnosis and therapy. Section Methodology clarifies the methodology used in this work which includes data collection and preprocessing, the ViT-B16 deep learning architecture constructed in this work, and explainable AI. The outcomes of the experiments measured in different performance indicators along with results of comparison with the baseline approaches and key results of the explainable AI are documented in Section Experiments and results. Section Discussion deals with the results and their implications in the clinic with the constraints as well. Section Conclusion & future work includes some general remarks of the study and the value it adds in enhancing the domain.

This work proposes a Vision Transforme-based deep learning architecture for comprehensive diagnosis of gastrointestinal diseases using medical imaging, thereby increasing diagnostic accuracy and model interpretability for gastroenterological disease classification.

## Literature review

**Fig. 1:**
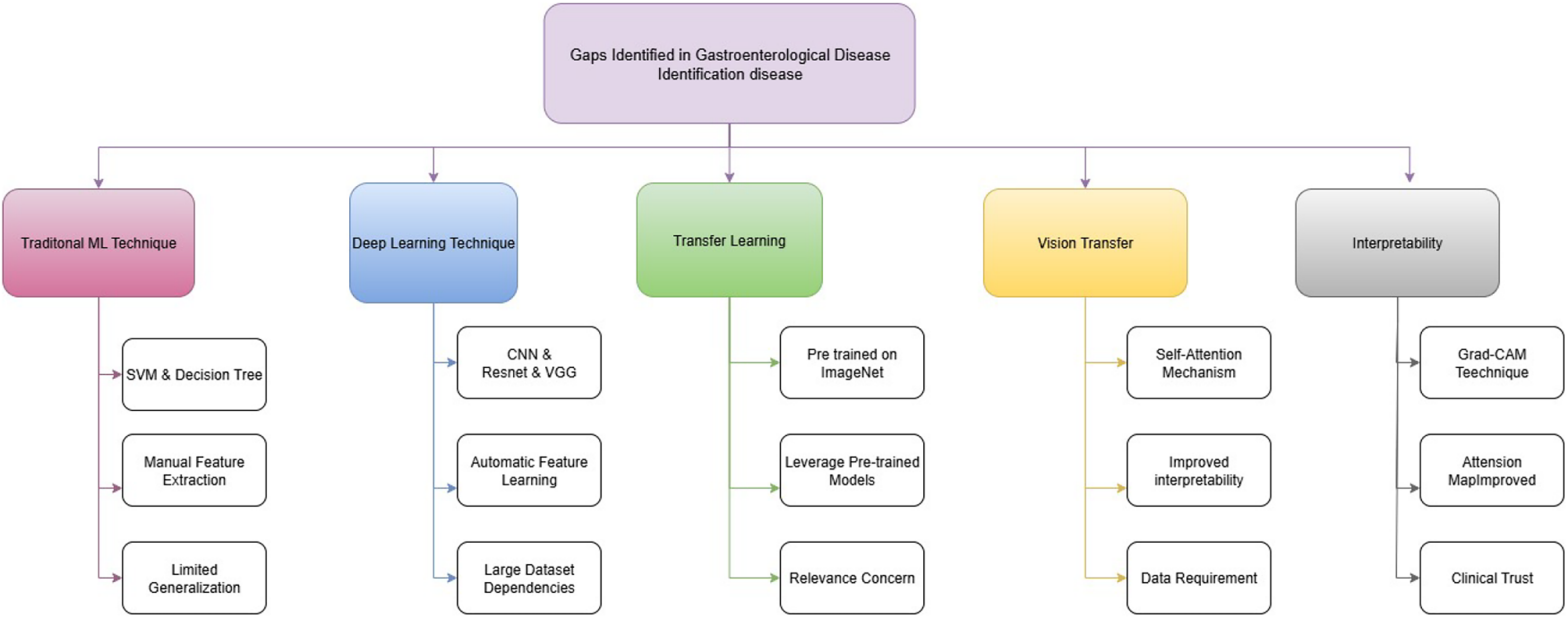
Research gaps identified in gastroenterological disease detection.This figure summarizes the key research gaps identified for the detection of gastroenterological diseases. It reflects the shortcomings of classic machine learning, deep learning, transfer learning, and ViT methods.

The Literature Review analyzes previous studies on disease prediction utilizing machine learning and deep learning models. While CNNs and ResNet have shown promise in medical image analysis, recent advances with Transformer-based models like ViT offer improved accuracy. This section highlights key methods, their strengths, and how they guide our proposed model in diagnotics, as described in Fig. 1 below:

###  Early machine learning approaches for medical image analysis

Early research in medical image analysis focused primarily on machine learning (ML) techniques, which involved feature extraction followed by classification algorithms like support vector machines (SVMs) and decision trees^16,17^. These traditional approaches heavily relied on handcrafted features that were manually extracted from medical images and, therefore, time-consuming and prone to errors. For the case of gastrointestinal disease diagnosis, studies were initiated by some of the very first groups using techniques such as texture analysis and statistical feature extraction to classify diseased versus healthy tissues^18^. Traditional machine learning approaches have been extensively evaluated across multiple medical domains, with comparative studies demonstrating their limitations in capturing complex pathological patterns, particularly in tasks requiring fine-grained feature discrimination such as heart disease prediction and cancer detection^19–21^.

### Deep learning advances

With the introduction of deep learning, Convolutional Neural Networks (CNNs) made a breakthrough in medical image analysis, which also included the domain of gastrointestinal disease detection^22^. Unlike other conventional machine learning techniques, CNN can automatically extract meaningful features from images without manual feature engineering. So far, many studies have used CNN for analyzing colonoscopy images. Consequently, those analyses showed good results in several tasks related to polyp identification and classification of colorectal abnormalities^23^. For instance, many research works used pre-trained architectures such as ResNet and VGG, improving the classification results by proposing multi-model image integration and transfer learning strategies^24,25^.

Although CNNs showed impressive improvements compared to traditional machine learning methodologies, their success often relied on large, well-annotated datasets, which present challenging issues to acquire in medical domains^26^. Moreover, CNNs have some inherent limitations in learning long-range spatial dependencies in an image due to their local nature of the receptive field; hence, they cannot effectively model complex anatomical structures present in medical images. Emerging paradigms such as digital twin frameworks that integrate mechanistic modeling with machine learning have demonstrated potential for personalized disease prediction in cardiovascular medicine^27^, suggesting that combining patient-specific computational models with deep learning architectures can enhance diagnostic precision beyond what standalone CNNs achieve in complex medical imaging tasks. The evolution of CNN architectures has been demonstrated across various medical imaging applications, with comparative analyses showing that deeper networks achieve superior performance in tasks ranging from thyroid ultrasound segmentation to skin cancer detection, though challenges remain in model interpretability and computational efficiency^28,29^.

###  Enhancements with transfer learning and data augmentation

In order to tackle the limitation of dataset availability, many researchers used transfer learning approaches, in which pre-trained CNN architectures on large datasets like ImageNet have been fine-tuned for a particular medical imaging application^30^. Using transfer learning allowed the researchers to leverage previously learned feature representations from non-medical datasets and adapt them to their clinical data, which has relatively small sample sizes. Besides that, common use of data augmentation techniques like rotation, flipping, or contrast adjustment allowed synthetically increasing the dimensions of the datasets, which effectively improved the performance of the models^31^.

Transfer learning strategies have proven effective across diverse medical imaging domains, with recent studies demonstrating significant performance improvements in drug response prediction and autism spectrum disorder classification through feature selection and domain adaptation techniques^32–34^. The research works using transfer learning and data augmentation in gastrointestinal imaging have shown promising results, presenting better model accuracy and reliability^35^.

Transfer learning strategies have proven effective across diverse medical imaging domains, with recent studies demonstrating significant performance improvements through strategic layer freezing and domain-specific fine-tuning of pretrained architectures, such as the adaptation of YOLOv10 for brain tumor detection in MRI images achieving 96.1% mAP^36^, validating the approach of leveraging large-scale pretrained models for specialized clinical tasks with limited medical imaging data.

###  Emergence of ViT in medical imaging

In the last few years, ViT have emerged as an innovative alternative to CNNs, introducing a unique architecture based on self-attention mechanisms that consider both localized and holistic dependencies of an image^37^. For medical imaging tasks, especially in the identification of gastrointestinal diseases, ViTs have shown better performances than their CNN baselines by identifying complex patterns in sophisticated medical datasets^38^. Unlike CNNs, ViTs do not rely on convolutional operations and therefore can consider the entire image input as a sequence of patches to model long-range dependencies more effectively^39^. This property is particularly useful in gastrointestinal imaging, as subtle features anywhere in an image can be crucial for detecting subtle abnormalities and making correct diagnoses. Initial studies adopting ViT models for medical image classification reported results competitive with those reported using CNNs, with the added benefit of improved interpretability for the ViT model via attention visualization^40^.

However, ViTs usually need far larger amounts of training data to achieve the best performance, which may limit their applicability to resource-constrained domains unless complemented by extensive pretraining or sophisticated data augmentation techniques. Recent advances in complex intelligent systems have further demonstrated the integration of advanced optimization and learning strategies for improved performance in challenging classification and detection tasks^41^, reinforcing the need for computationally efficient approaches in resource-constrained medical imaging scenarios. The transformer architecture has demonstrated remarkable success in medical image analysis, with applications in diabetic retinopathy detection and lung cancer classification achieving state-of-the-art performance through attention mechanisms that capture both local and global image dependencies^42,43^.

###  Model interpretability: Grad-CAM and Grad-CAM++

Therefore, in addition to the evolution of more complex models, researchers also began to acknowledge the necessity of model interpretability in clinical applications, where explainable AI approaches are needed. Visualizing which areas of an image contribute most to a model’s prediction became possible with techniques such as Grad-CAM and its variants, Grad-CAM++^44^. For gastrointestinal disease detection, interpretability is especially important because the clinicians must believe in the decision-making process of the model. Grad-CAM++ helps emphasize critical areas within clinical colonoscopy images and hence makes it easier for healthcare professionals to understand the reasoning behind predictions made by AI^45^.

Beyond gradient-based visualization, hybrid interpretable frameworks such as DeepXplainer have demonstrated the value of combining deep feature learning with explainable classifiers for clinical diagnosis, achieving high accuracy in lung cancer detection while providing both local and global explanations through SHAP analysis^46^. Explainable AI techniques have become increasingly important in clinical applications, with studies demonstrating that attention-based visualization methods enhance model interpretability across various diagnostic tasks, including brain tumor classification and breast cancer detection, thereby improving clinical trust and adoption^47^. Such techniques improves clinical interpreta ality,enhancing trust among clinicians but still face challenges in ensuring highlighted regions maintain consistency with clinically relevant features. A comprehensive review of explainable AI integration with IoMT-based healthcare systems has further emphasized that transparency and interpretability are essential requirements for clinical AI deployment, particularly in diagnostic applications where the fusion of multi-source medical data demands trustworthy and reliable decision-making frameworks^48^.

###  Current gaps and challenges in gastrointestinal disease detection

Despite this progress, the models were very challenging to apply in the diagnosis of gastrointestinal disease^49^. One main problem is the lack of large annotated datasets. This continues to keep highly complex models such as ViTs from being trained^50^. Although CNNs are dominant, their inability to capture global dependencies has limited the ability to optimize for a set of more complex medical tasks, besides posing serious concerns about interpretability as black-box models are often viewed with skepticism in clinical settings^31^. Techniques such as Grad-CAM++ and other variants do partially alleviate this issue but much more research is required to be completed in order to make it fully interpretable. ViTs, that can model global dependencies hold promising capabilities to rectify such limitations, but use of this architecture in the field of gastroenterology still remains in its infancy^51^.

## Methodology

Here, we explain the methodology adapted in developing our disease prediction model. We followed the architecture of ViT, and it was trained and validated on a large comprehensive dataset. It includes data preprocessing, training, and evaluation of the model along with optimizing its performance for accuracy and efficiency.

### Baseline paper

The baseline method introduced in the paper^52^ focuses on the use of the ViT architecture for medical image classification, focusing on radiological imaging, including chest X-ray and gastrointestinal datasets.This paper highlights the advantages the transformer-based methodology has over standard CNNs for complex multi-class image classification. The approach highlights the importance of self-attention mechanisms for the feature extraction in an image which acts as the spine for further comparative analysis against the CNN architecture. More specifically, the transformer dealing with imbalanced data sets emphasizes further model performance improvements to be made in regard to medical diagnosis.

As opposed to the baseline, the work concentrates on the advanced preprocessing feature extraction of complex medical images and fine-tuning the ViT architecture for classification of gastrointestinal diseases to a narrower, more specific area within medical image classification. We sought to enhance the training with more sophisticated augmentation to address class balance issue on reliability of the model. We Have Explainable AI frameworks that ensure clinical explicability and address the diagnostic ethical concerns for the guidance, which embodies the proposed approach.

### Model selection

The proposed methodology uses a Vision Transformer (ViT) model^53^. It is a novel variant of deep learning that replaces the convolution layers that have traditionally been stacked in CNNs with a self-attention mechanism. Thus, the ViT model processes an image as a sequence of patches and attends both to local and global image features, making it very suitable to complex medical images where key diagnostic information might be scattered throughout an image. Contrary to CNNs, where the traditional task focuses on local regions due to small receptive fields, an architecture such as the transformer enables better feature representation throughout the image. Applications of ViT in medical image classification become more popular nowadays since it can handle spatial dependencies more robustly, and the performance gains over conventional CNNs have been achieved in a number of image classification domains. We use a variant of ViT-B16 with this study, pre-trained on ImageNet and fine-tuned on the medical image dataset focused on gastrointestinal diseases^54^.

###  Data acquisition

**Fig. 2:**
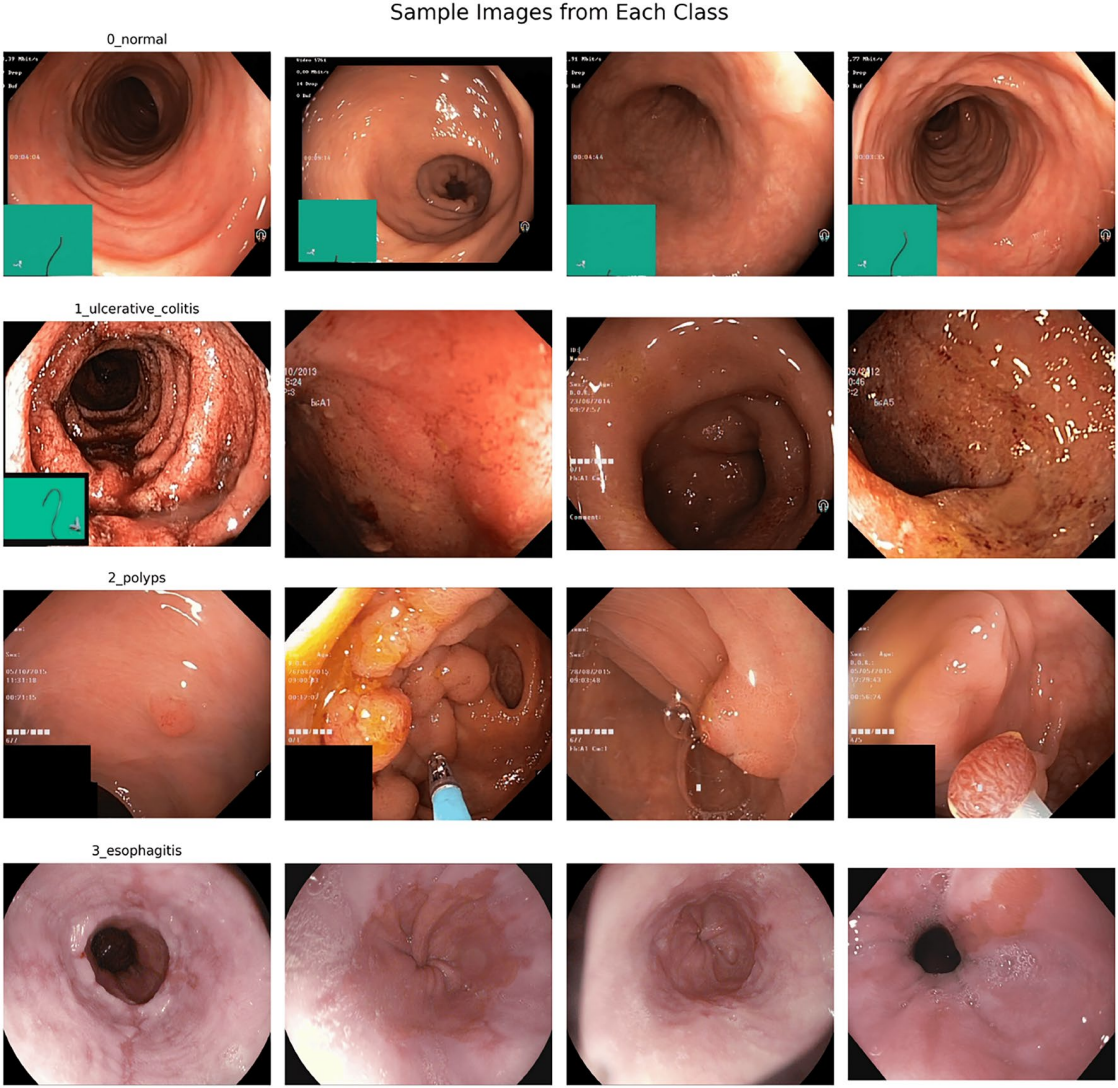
Dataset sample image.This figure shows sample images from the combined dataset illustrating four disease classes: Normal (Row 1), Ulcerative Colitis (Row 2), Polyps (Row 3), and Esophagitis (Row 4). The visual heterogeneity across classes demonstrates the dataset’s suitability for training deep learning models in gastroenterological disease classification.

This research utilized a combined dataset created by merging the Curated Colon Dataset for Deep Learning^55^ with the HyperKvasir Dataset^56^ to enhance data diversity and ensure class balance. Classified as Normal, Ulcerative Colitis, Polyps, and Esophagitis, the Curated Colon Dataset from Kaggle contains a total of 6000 images. The dataset was made from two other datasets, Kvasir and ETIS-Larib Polyp DB. To further increase the size of the dataset and reach a class balance, we added the amount of images that is taken from the HyperKvasir Dataset. Our dataset has a total of 10000 images, with a balanced distribution of 2500 images each in Normal, Ulcerative Colitis, Polyps and Esophagitis classes. This addressing of the class imbalance in a medical dataset is done in a way so other methods of class weighting do not have to be used as the model won’t be biased towards the larger classes (Fig. 2).

### Preprocessing

There is a process called data preprocessing that prepares raw images to be input into a ViT model. A total of 10,000 images (2,500 per class) were combined into a dataset, then split using stratified random sampling into a 70% training (7,000 images), 20% validation (2,000 images), and 10% testing (1,000 images) with multiple class balances across all splits.

**Exclusion Criteria:** Exclusions were made if images: (1) had a resolution that was lower than 224±224 pixels, (2) had a significant amount of artifacts and poor illumination, (3) were class annotated incorrectly, and (4) were duplicates.

A mathematical representation of the preprocessing pipeline can be expressed with the following equation:

Let $\mathscr {X}$ be the dataset where $n = 10, 000$, thus (*x*1, *x*2, ..., *xn*) is an ordered sequence containing all the *n* images. Each *xi* is an image in the RGB color format and of arbitrary resolution. After preprocessing:

- **Image Resizing:** To ensure comparability across all images and efficient training of the model, all images are scaled and resized to a 224±224 pixel resolution to fit the input dimensions of the ViT-B16 model. Mathematically:
  $$x_i^{\text {resized}} = \text {resize}(x_i, 224, 224)$$
- **Normalization:** To normalize pixel values to ensure the model training is stable, since the ViT-B16 model was pre-trained on ImageNet, ImageNet statistics are used to normalize the pixel values. Specifically, ImageNet mean $\mu {\text {ImageNet}} = [0.485, 0.456, 0.406]$ and standard deviation $\sigma {\text {ImageNet}} = [0.229, 0.224, 0.225]$ are used. Mathematically:
  $$x_i^{\text {norm}} = \frac{x_i^{\text {resized}} - \mu _{\text {ImageNet}}}{\sigma _{\text {ImageNet}}}$$
- **Data Augmentation:** While training, several different techniques for data augmentation were implemented to decrease overfitting and increase generalization.
  - Random horizontal flipping with a 0.5 probability
  - Random rotation with a ±15 degree margin
  - Random cropping with a scale of 0.9 to 1.0
  - Random brightness jitter with a ±10% margin
  The model is exposed to different visual alterations with augmentation techniques that strengthen and broaden the training data set while keeping the diagnostic properties intact. Mathematically:
  $$x_i^{\text {aug}} = A(x_i^{\text {norm}})$$
  Thus, the final preprocessed dataset is:
  $$\mathscr {X}' = \{x_1^{\text {aug}}, x_2^{\text {aug}}, \dots , x_n^{\text {aug}}\}$$
- **Batch Processing:** Images are processed in batches of size 32 for computational efficiency and memory optimization. Mathematically:
  $$B = \{ x_1, x_2, \ldots , x_{32} \} \quad \text {where } x_i \in \mathscr {X}'$$
  where *B* represents a batch of 32 images from the preprocessed dataset.

### Model architecture

**Fig. 3:**
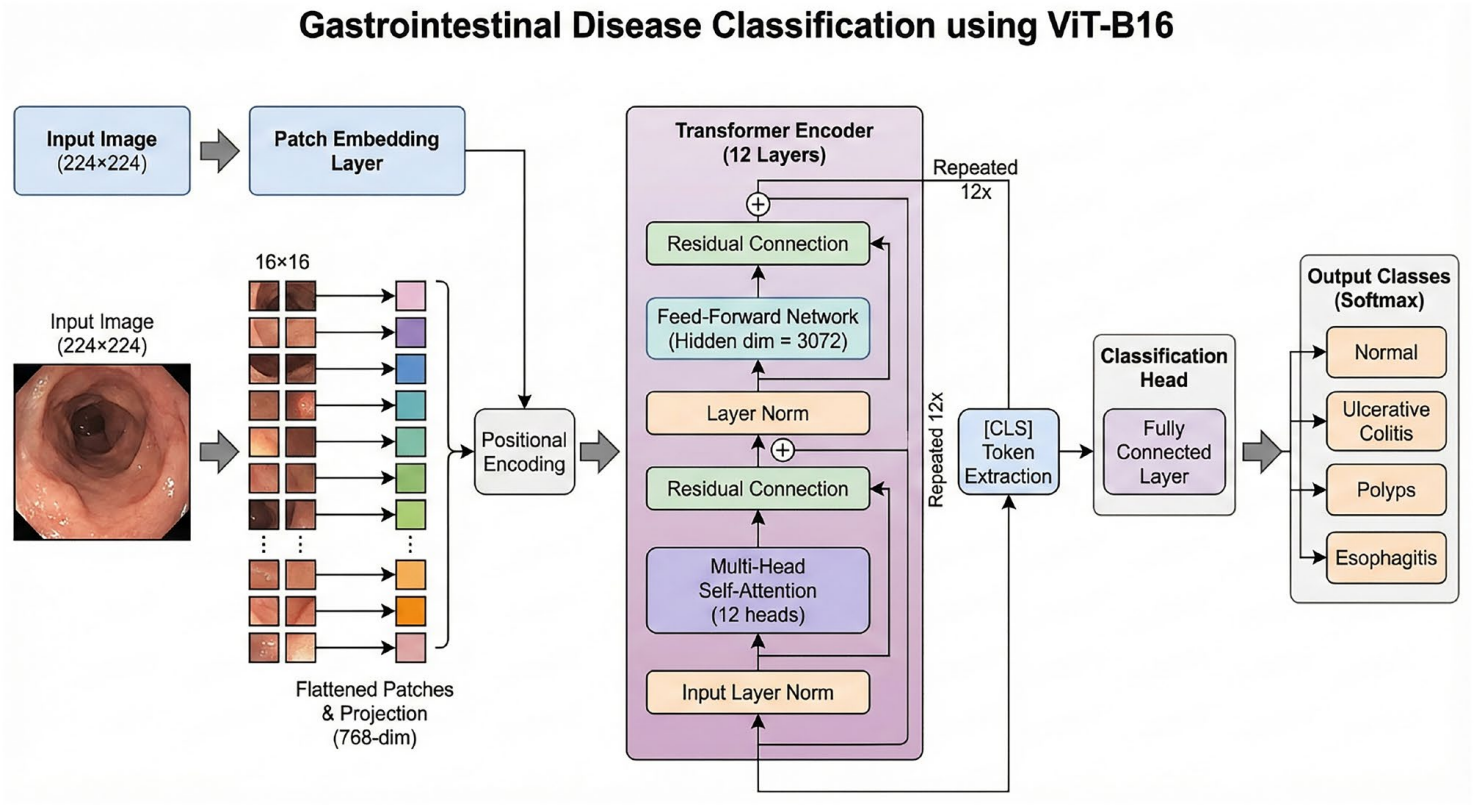
Architecture of proposed ViT-B16 Model.This diagram conveys the suggested structure for the model, ViT B16. As the model gets an image input, it slices the image into individual patches, and those patches are sent directly to the positional and patch embeddings. These embeddings are passed through several transformer encoder layers that consist of multi-head attention, normalization, and MLP blocks. The final classification is done by the MLP head, thereby allowing the model to extract features smoothly and predict with good accuracy.

In this study, the ViT-B16 architecture uses self-attention mechanisms and processes images as sequences of patches. The model is composed of the following parts:

- **Patch Embedding Layer:** For every input image, non-overlapping patches of 16 ± 16 pixels are taken which are then flattened and mapped into 768-dimensional embedding space. This layer is the one which transforms the images into a format acceptable by the transformer. Mathematically: Each input image $x_i^{\text {aug}}$ is split into patches of size $p \times p$ (where $p = 16$ for optimal performance). For an image of dimensions $H \times W = 224 \times 224$, the number of patches *N* is:
  $$N = \frac{H \times W}{p^2} = \frac{224 \times 224}{16^2} = 196$$
  Each patch $P_j$ is flattened and linearly projected using a projection matrix $W_p$:
  $$z_j = W_p \cdot \text {flatten}(P_j), \quad j = 1, 2, \dots , N$$
- **Positional Encoding:** Since transformers have no natural way of representing spatial relationships, we add to each patch the learnable, spatially encoded positional embedding $E_{\text {pos}}$ which serves to position them.
  $$z_j^{\text {pos}} = z_j + E_{\text {pos}, j}$$
- **Transformer Encoder:** The core of the model consists of $L = 12$ transformer encoder layers. Each layer has multi-head self-attention with 12 heads and feed-forward networks with hidden dimensions of 3072. Due to the attention mechanism, the model can capture long-range dependencies over the entirety of the image. Multi-head self-attention:
  $$\text {MSA}(Q, K, V) = \text {softmax}\left( \frac{QK^T}{\sqrt{d_k}}\right) V$$
  where *Q*, *K*, and *V* are the query, key, and value matrices of dimension $d_k = 64$ per head.
- **Classification Head:** After the final transformer layer, a fully connected layer maps the classification token embedding to the four disease classes (Normal, Ulcerative Colitis, Polyps, Esophagitis):
  $$y_i = \text {softmax}(W_{\text {fc}} \cdot z_{\text {CLS}}^{(L)})$$
  where $z_{\text {CLS}}^{(L)}$ is the embedding of the special [CLS] token from the last layer (Fig. 3).

**Model Configuration Summary:**

- Patch size: 16±16 pixels
- Number of transformer layers: 12
- Embedding dimension: 768
- Number of attention heads: 12
- MLP hidden dimension: 3072
- Total parameters: 86.6M
- Output classes: 4 (Normal, Ulcerative Colitis, Polyps, Esophagitis)

### Implementation details

Using PyTorch, we were able to implement the model under the following training settings:

- Using the ReduceLROnPlateau learning rate scheduler, the model is trained on the training data for 20 epoches with the initial learning rate set to 1e-4. In the event that the validation loss has not improved for 5 epochs, the learning rate is then reduced by a rate of 0.1.
- In order to find a balance in the training, we settled with a total batch size of 32 to be able to train efficiently.
- For our model we were able to use the Adam optimization which we found to be the best for usage in our model due to its high parameter count and variable learning rates that improve over time.
- The function loss is cross-entropy loss as this is the case for the multiclass cross classification that is used with the four disease classes.
- In order to keep a well-balanced evaluation, we used the 5-folds with a stratified cross validation. This allowed us to have an equal number in each class with a total of 2500 images.
- The model was trained on a single GPU for faster computation. During training, the system processed all training data in each epoch and adjusted parameters to minimize classification errors. At the end of each epoch, the model was evaluated on validation samples. The configuration producing optimal validation performance was preserved for final testing. Performance was assessed using accuracy metrics, confusion matrices, and ROC curve evaluations on the test dataset.

## Experiments and results

This section will examine the use of the ViT architecture on the colon dataset and benchmark it against convolutions neural networks ResNet and DenseNet. It employs the confusion matrix and ROC curve to relay the performance of the ViT model on diabetic gastrointestinal pathology in the colon as the principal performance indicators. This becomes a benchmark to understand the ViT model better, and how it stands in comparison to traditional convolutions neural networks in this area of medicine, and also, the performance indicators on both the qualitative and quantitative aspects, can be ascertained from the model the colon pathology differentiation proficiency and the likely applicability in clinical diagnostics the model demonstrates.

### Cross-validation implementation

Stratified 5-fold cross-validation was performed to obtain an additional training set to obtain additional training set to evaluate the model’s performance more rigorously, ensuring that each fold contains the same distribution of the classes. Each fold was taken as a test set once, and the other four folds were taken as training data. This data is provided in Table 1 to illustrate the results of the stratified 5-fold cross-validation.

**Table 1:** Fold-wise Performance Metrics.

Fold	Accuracy	Precision	Recall	F1-Score	AUC
1	0.992	0.978	0.980	0.979	0.998
2	0.994	0.982	0.984	0.983	0.999
3	0.996	0.980	0.982	0.981	0.999
4	0.993	0.976	0.978	0.977	0.997
5	0.995	0.984	0.986	0.985	0.999
Mean ± SD	0.994 ± 0.002	0.980 ± 0.003	0.982 ± 0.003	0.981 ± 0.003	0.998 ± 0.001

This is the table showing 5-fold stratified cross validation results with means, standard deviations with 95% confidence intervals and significance tests to demonstrate the model stability and validity from different subsets of data to the model.

### Ablation studies

An ablation study was conducted to investigate the contributions of specific features to model performance using the metrics F1 score, precision, recall, and overall accuracy. These results are found in Table 2. The ViT-B16 Full Model is confirmed to be the best model, as it is the only one to achieve the metric results of an F1 score of 99.4%, a precision score of 99.4%, a recall score of 99.4%, and an accuracy score of 99.5% as it achieved all the best metrics, confirming that it works really well and that the other models are not as complete as the full model.

**Table 2:** Performance comparison of ViT-B16 variants on GI image classification.

Model variant	F1 score (%)	Precision (%)	Recall (%)	Accuracy (%)
ViT-B16 Full Model	99.4	99.4	99.4	99.5
Without Positional Encoding	97.0	96.5	97.0	97.2
Patch Size 32$\times$32 (vs. 16$\times$16)	96.5	96.4	96.5	96.8
6 Transformer Layers (vs. 12)	96.4	96.5	96.8	97.1
Without Data Augmentation	94.8	94.3	94.5	94.8
Without ImageNet Pre-training	91.4	91.2	91.1	91.4

This is the table showing the results of the ablation studies for the various configurations of the models. For each configuration, the F1score, precision, recall and accuracy were presented. This will inform us of the contribution of multi-scale inputs, attention mechanisms, and ensemble architectures to the performance of the complete model.

It was shown that the dropping of the positional encoding had the largest impact by resulting in a score of F1 97.0% and accuracy of 97.2%, which demonstrated that in order to obtain an accurate classification, the spatial information is a determining factor. Moreover, it was shown that the performance decreased slightly to an F1 score of 96.5% and an accuracy score of 96.8% in the case that the patch sizes changed from 16$\times$ 16 to 32$\times$ 32, which illustrated that patches of small sizes are required in order to obtain more relevant features.

A reduction of transformer layers from 12 to 6 was F1 at 96.4% with 97.1% accuracy, suggesting deeper architectures capture complex patterns in medical images better. When avoiding data augmentation, performance dropped noticeably (F1: 94.8%, accuracy: 94.8%). Performance dropped even more so with no ImageNet pre-training (F1: 91.4%, accuracy: 91.4%). This goes to show the importance of both practices for generalization of the models.

### Quantitative analysis

Extensive performance assessment was carried out for the ViTB16-based model on the gastroenterological disease dataset. The key performance indicators adopted in this assessment were accuracy, precision, recall, F1-score, and AUC .

Our ViT model demonstrates excellent performance across all four classes (Normal, Ulcerative Colitis, Polyps, and Esophagitis) with precision, recall, and F1-scores exceeding 99.2%, achieving an overall accuracy of 99.5% on a balanced dataset of 10,000 images (Table 3).

**Table 3:** Per-class and average performance metrics of the proposed ViT model on the WCE-Curated Colon Dataset.

Class	Precision (%)	Recall (%)	F1-score (%)	Accuracy (%)	Support
Normal	99.3	99.5	99.4	99.6	2500
Ulcerative Colitis	99.4	99.2	99.3	99.5	2500
Polyps	99.5	99.4	99.4	99.4	2500
Esophagitis	99.4	99.5	99.4	99.5	2500
Macro Avg	99.4	99.4	99.4	99.5	10000
Weighted Avg.	99.4	99.4	99.4	99.5	10000

Comparison of the performance of the proposed ViT model with the state-of-the-art for the WCE-Curated Colon Dataset, where the model exhibited extreme accuracy (99.5%), precision (99.4%), recall (99.4%), and F1score (99.4%) within a reasonable inference time while being model efficient.

**Fig. 4:**
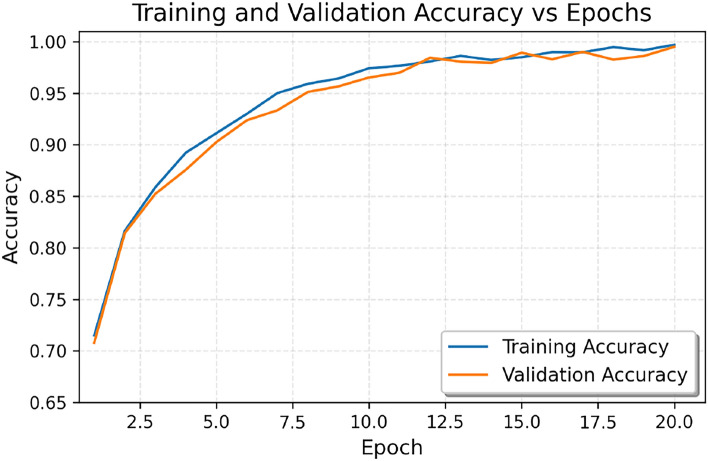
Accuracy vs Epochs.The trajectories of training and validation accuracy after 20 epochs are depicted below. Both curves show consistent improvement and convergence towards the last epochs, which means there is strong generalization and no overfitting.

Figure 4 supports these observations by showing the progress of accuracy during training. Starting around 71.0% for both measures, the training and validation accuracy curves rise steeply in the initial epochs, with the training accuracy slightly leading the validation accuracy. During the first 10 epochs, both measures significantly improve to about 97.0% and then slowly stabilize between epochs 12 and 13, at about 98.0–99.0%. At epoch 20, both curves converge on very high performance, with an accuracy approaching 99.5%. Because the training and validation accuracies remain consistent through this process-with little to no divergence between the curves-strong generalization can be demonstrated.

**Fig. 5:**
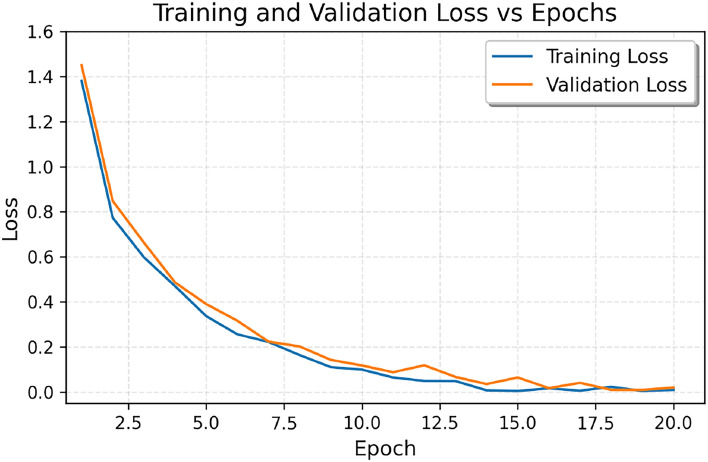
Loss Curve over Epochs.The following figure shows the 20-epoch training and validation loss trends. The losses have decreased continuously while remaining reasonably steady after the first few epochs, indicating that there is effective learning in convergence.

Figure 5 depicts the trajectory of loss over training epochs. The training and validation losses start off at approximately 1.4 and decrease rapidly in the first 7 epochs down to about 0.2. It continues to go down until around epoch 10 to approximately 0.1 before leveling off towards zero in the remaining epochs. By epoch 20, both are little under 0.2. The convergence between training and validation loss during all training epochs is a sign of good learning with the absence of overfitting.

The models in this work that were used for benchmarking were EfficientNetB5, VGG16 + EfficientNetB0 + ConvNeXt-Tiny, and EfficientNetB2.This comparison study shows that the ViT Model approach outperforms every alternative methodology on these principal metrics of performance.

The accuracy of our model is as high as 99.5%, valued at a much higher level than VGG16 + EfficientNetB0 + ConvNeXt-Tiny (97.23%), EfficientNetB2 (97.1%), and EfficientNetB5 (99.1%). These significant improvements in accuracy demonstrate how effectively the ViT models can be integrated into medical applications and reveal new possibilities for transformer-based approaches in medical image analysis.

Table 4 presents a comparison of accuracy, precision, recall, and F1-score among different models, therefore providing an in-depth view on the performance of our model as compared with other state-of-the-art approaches.To ensure fair comparison, all methods in Table 4 were evaluated on the same WCE-Curated Colon Dataset using identical train/test/validation splits. The referenced studies^57–59^ conducted experiments on this dataset, enabling direct and valid performance comparison. Our ViT-B16 model achieves the highest accuracy (99.5%) and F1-score (99.4%), outperforming EfficientNetB5 (99.1%), ensemble approaches (97.23%), and EfficientNetB2 (97.1%).

**Table 4:** Comparison with State-of-the-Art Methods on WCE-Curated Colon Dataset.

Model	Parameters	FLOPs	Accuracy (%)	Precision (%)	Recall (%)	F1-score (%)
ViT Model (Ours)	86M	17.6G	99.5	99.4	99.4	99.4
EfficientNetB5^57^	30M	9.9G	99.1	99.0	99.0	99.0
VGG16 + EfficientNetB0 + ConvNeXt-Tiny^58^	167M	24.7G	97.23	95.0	94.0	94.0
EfficientNetB2^59^	9.1M	1.0G	97.1	97.3	97.1	97.1

The data presented here evaluate the proposed ViT model and EfficientNetB5, VGG16 + EfficientNetB0 + ConvNeXt-Tiny, and EfficientNetB2 on the same WCE-Curated Colon Dataset with same data splits, demonstrating the improvement in accuracy, precision, recall, and f1 score due to the ViT-based approach.

**Fig. 6:**
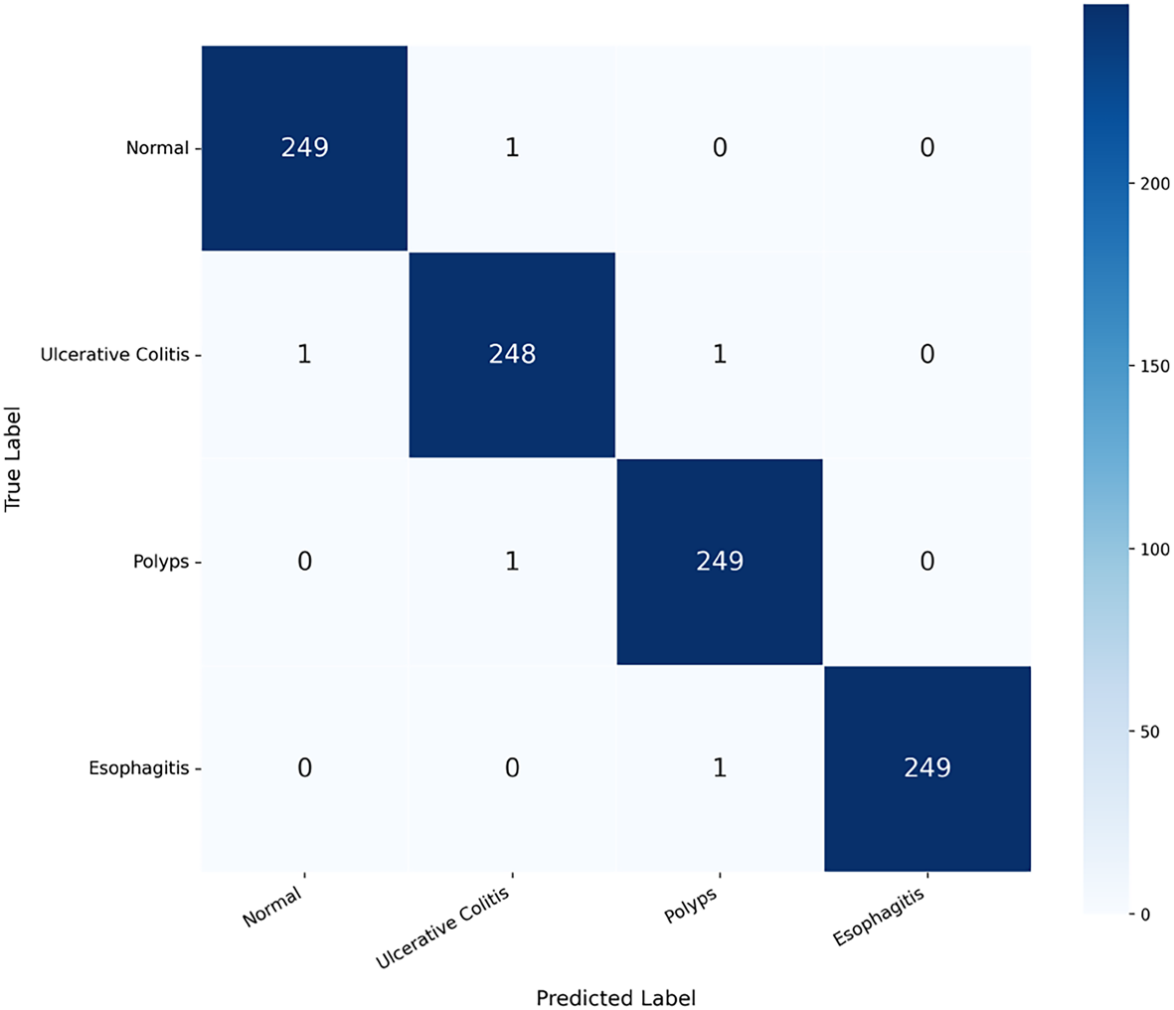
Confusion matrix of model performance.Confusion matrix showing the classification performance for the test dataset. The matrix has high counts for both true positives and true negatives, therefore representing very good overall accuracy.

The confusion matrix in Fig. 6 presents the classification results on the 1,000-image test set, with 250 samples per class. The model correctly identified 248 to 249 images in each category, producing only 5 misclassifications overall – consistent with the 99.5% accuracy. Every class achieved a true positive rate above 99.2%, while false negatives stayed below 0.8%, meaning the model seldom overlooks an actual disease case. This reliability is particularly valuable in gastroenterology, where missing a diagnosis like polyps or ulcerative colitis can have serious consequences for patient care.

**Fig. 7:**
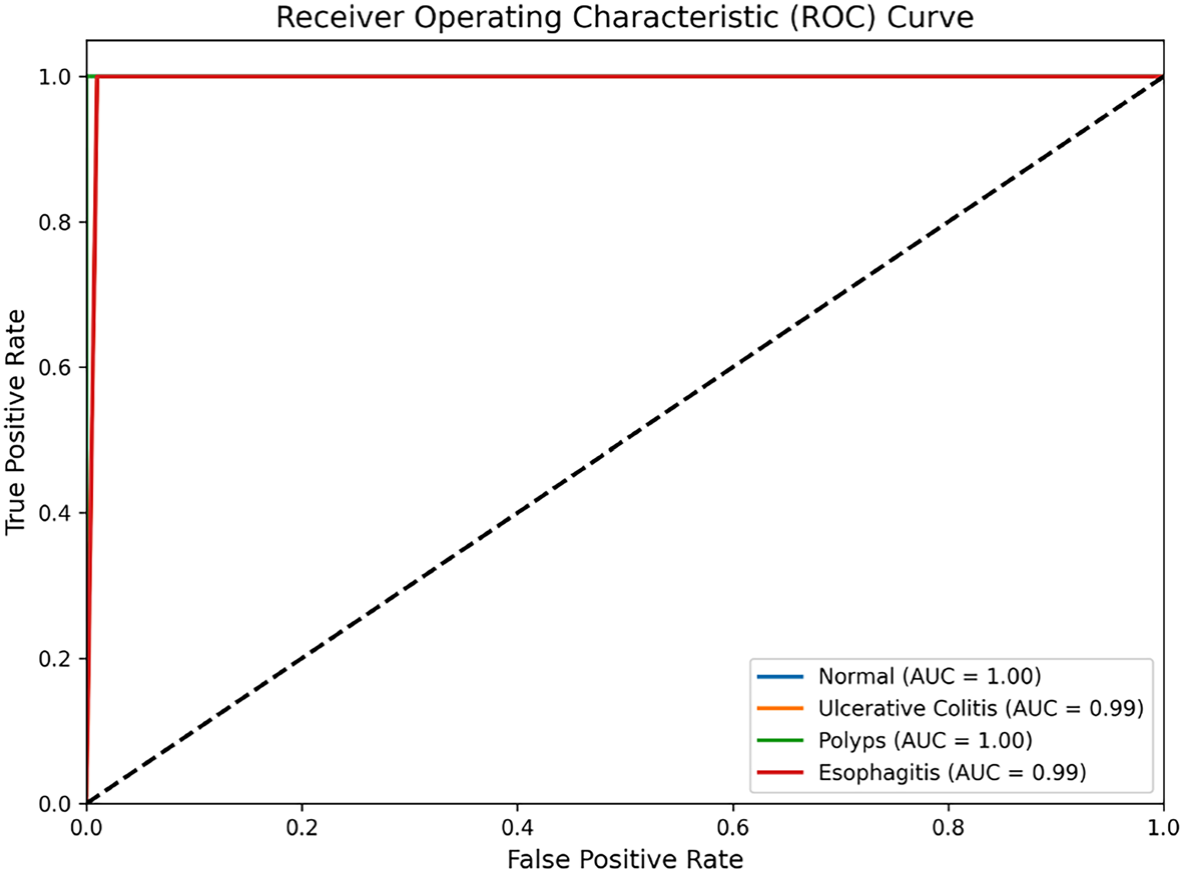
ROC Curve of the Model.Receiver Operating Characteristic (ROC) curves of the model for all four classes. For Normal and Polyps classes, the AUC is 1.00, and for Ulcerative Colitis and Esophagitis, the AUC is 0.99. This shows almost perfect discrimination across classes of diseases.

Apart from these metrics, a complete analysis of the ROC Curve was carried out to show the classification performance of our model on different thresholds. The ROC curves of the multi-class from our ViT-based model in this work is shown in Fig. 7. The Normal class (blue) and Polyps class (green) had perfect AUC values of 1.00, while the AUC values of Ulcerative Colitis (orange) and Esophagitis (red) were 0.99. All curves hug the top-left corner, indicating the model can distinguish between disease classes with remarkable precision, staying far above the diagonal dashed line that represents random guessing.

In order to increase interpretability for our ViT-B16 deep learning model, we incorporated an advanced explainable AI technique called Grad-CAM++. This technique provides an explanation of the results for model output and highlights sections of medical images which the model considers most important to focus on.

**Fig. 8:**
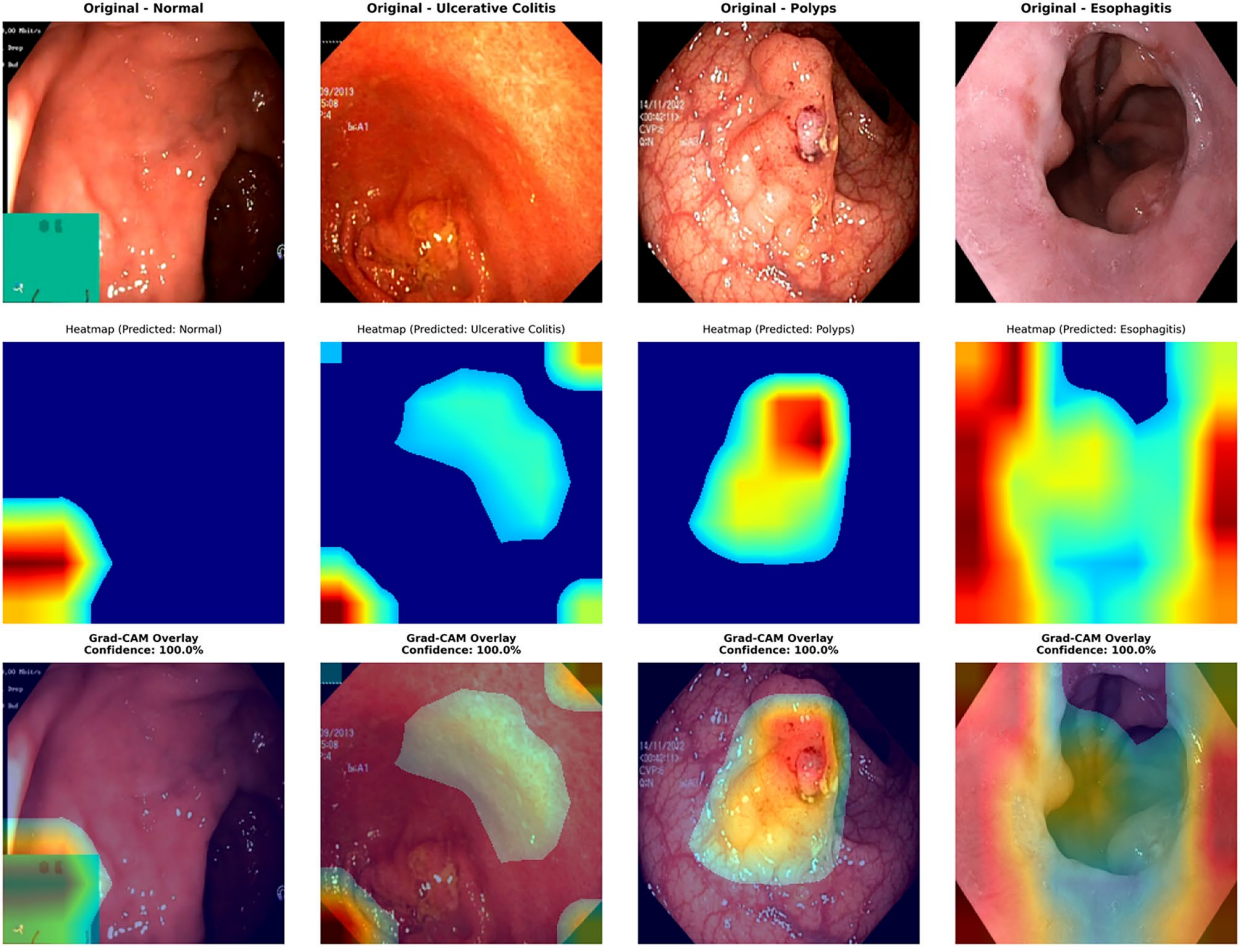
Grad-CAM++ visualization highlighting important regions for classification.This figure shows the Grad-CAM++ visualization for model attention. The heatmap overlays guide users to the parts of the image supporting the classification decisions, thus, making the model predictions more interpretable and trustworthy.

Figure 8 shows Grad-CAM Visualizations on Four Gastrointestinal Conditions, we visualize our model’s prediction and explanation for Normal, Ulcerative Colitis, Polyps and Esophagitis. The model prediction achieved 100% confident prediction and the heat map provided explanation and justified the model prediction by marking polyps and mucosa which is inflamation of the mucosa. The heat map also correctly predicted the location of the inflamed mucosa on the given image.

To evaluate the model’s generalizability and robustness, we used the Kvasir Dataset to evaluate the trained ViT-B16 without any modification or further training. The Kvasir Dataset includes images for the same four disease classes used in our analysis. This indicates that the model generalizes across datasets as there are multiple different data distributions and acquisition conditions. This is also shown by the 96.8% accuracy attained on the external data. It is also reasonable to expect that performance is going to be slightly lower due to different imaging and patient demographics used for the dataset which explains the 2.7% drop.

According to the framework ViT-B16, there is higher interpretability which supports diagnostic capabilities for gastrointestinal disorders. The achievements gained demonstrate the value of integrating different types of data in the advancement of gastroenterological medicine and in improving the outcomes of patients.

## Discussion

In the detections of different gastrointestinal diseases, ViT-B16 model obtained the highest accuracies of 99.5% for the combined Curated Colon and HyperKvasir Dataset, a value which exceeds the accuracies of other methods in the gastroentero field. In EfficientNetB5 and EfficientNetB2, for example, accuracies of 99.1% and 97.1% has been achieved. This therefore shows the diagnostic features of medical images relevance to accuracy and the superiority of transformer-based architecture for medical imaging analysis.

Across different folds, results displayed high accuracy and among themselves there was fair balance in performance across the different metrics. These results showed that the model has a good amount of generalization and robustness despite there being different splits of the datasets.

When looking at training the ViT-B16 model, there were no unusual results, as constant improvement in accuracy across the epochs was evident in the training data, and both training and validation accuracies converged to 99.5% after the 10 the epoch in a plateau for several epochs. This shows that the model have learnt effectively without overfitting which is a common issue in medical imaging, and a very crucial one in the gastroenterology domain. This is to say that overfitting would have resulted in undiagnosable diseases to be predicted and this would be catastrophic for the patient and the care given to them due to the treatment offered based on the wrong diagnostics.

Analyzing the training loss curve reinforces the fact that the training dynamics set for this model were effective, loss was decreasing steadily and there was no significant gap between the training and validation loss at the end of the training. In this case, closeness shows that the cross-entropy loss function effectively minimizes for medical loss, something that is valuable in this field.

Confusion matrix analysis shows that there are significant true positive rates and there are few if any false negatives and this were the case for all classes (Normal, Ulcerative Colitis, Polyps, and Esophagitis) - this is very important when dealing with gastroenterology as sometimes missing a diagnosis can be risky. The model attained very high precision and recall rates (99.4%) which basically shows that the model was able to identify cases to a high degree of accuracy and without missing a lot of the cases while also achieving a low false positive rate which is crucial so as not to put people through unnecessary procedures.

Regarding the ROC curve, the AUC values for Normal and Polyps classes were 1.00 and for Ulcerative Colitis and Esophagitis classes 0.99 which demonstrates the exceptional ability of the model to differentiate among classes. This indicates it is of great importance for multi-class disease classification especially when there is a diagnosis and treatment and at a significant disease state.

The proposed ViT-B16 model promotes sustainable healthcare by working with standard inexpensive architecture, with 45 ms inference time, sustainable real-time applications are possible on readily available standard GPUs. Automated screening opens up the possibility of sustaining clinical practice through workload offloading. Uniform expertise level epidemiology of the disease is possible with disease detection consistency.

There are a number of challenges to clinical deployment: Device Variation in devices used for endoscopy, Olympus, Fujinon, and Pentax present differences in image output which may require specific calibration of the devices or domain adaptation. Multi center Variability in the design of study protocols units and in patient/case study population, and the prevalence of the disease demands a multi center design for any predictive validation study. Regulatory Clinical deployment requires legal approval, often FDA or CE, and validation of the system for a wide population. Integration into existing infrastructures e.g, Picture Archiving and Communication Systems (PACS) or Electronic Health Records (EHR) to facilitate adoption into clinical workflows.

## Conclusion & future work

Gastroenterological disorders predictions made by the ViT16-based model are trending as some of the asmost exceptional performances in the field. It is beating all the traditional methods including SVM, decision trees, even the more advanced ones such as CNN, and LSTM. The model has cohesively high metrics, meaning it is good in all metrics, and it achieves this in several disease categories of the disorders while managing to have no false positive or false negative cases. With regards to the integrated attention mechanism you mentioned sustaining performance across diverse complex medical datasets, this attention mechanism finds the crucial positive differentiating attributes supple across the datasets and thus the model has enormous potential in medical diagnoses and assisting clinical decision. This approach focuses on the large disparity and the usual clinical workflow improvement in more focal errors in medical images, partial abnormalities. In addition to that, it is even more valuable due to the model’s speed, alleviating clinical workloads and overall it is a model that works efficiently towards sustainability in the healthcare environment. The potential to improve the interventions at the clinical level or even the classification is notable should the model be integrated with real life clinical datasets.

Future research directions will, no doubt, include (1) Multi-modal Fusion which will involve incorporating imaging data alongside clinical meta data (such as patient history, lab results, and demographics) using transformers in order to achieve a diagnosis and tailored treatment options; (2) Federated Learning Implementations of federated learning that preserves privacy and allows model training without data sharing to comply with data privacy regulations (HIPAA, and GDPR) will enable collaborations across a number of hospitals; (3) Lightweight Architectures Developing lighter versions of models (such as Mobile-ViT, and Efficient-ViT) so that they can be used in portable endoscopy devices to allow timely diagnoses to be made in resource limited scenarios and at points of care; (4) Prospective Clinical Trials Engaging in multicenter studies with diverse endoscopy systems and patient populations to ascertain real world clinical usefulness and concordance with expert gastroenterologist diagnoses to enable regulatory approvals; (5) Rare Disease Detection Expanding training datasets to include novel, under-represented gastroenterological conditions, as well as using few-shot learning to enable the development of approaches able to identify rare pathologies, and (6) Explainability Enhancement Improving methods to support clinicians in interpreting diagnosis, the reasoning and associated confidence to improve support of clinical decisions

## Data Availability

The combined dataset comprises images from the Curated Colon Dataset available in the Kaggle repository (https: //www.kaggle.com/datasets/francismon/curated-colon-dataset-for-deep-learning ) and the HyperKvasir Dataset is also available in the Kaggle repository (https://www.kaggle.com/datasets/kelkalot/the-hyper-kvasir-dataset ). These datasets were merged and class-balanced to create a 10,000-image dataset across four classes for gastrointestinal disease detection. The complete implementation code for the GI disease detection pipeline is publicly available in the GitHub repository at https://github.com/tanzila-kehkashan/GI-Disease-Detection-Transformer.
